# Use of a synthetic antimicrobial peptide (PA-13) alone or in combination with reduced gentamicin levels to control *Escherichia coli* contamination in stored boar semen

**DOI:** 10.14202/vetworld.2026.771-781

**Published:** 2026-02-26

**Authors:** Krittika Keeratikunakorn, Panida Chanapiwat, Ratchaneewan Aunpad, Natharin Ngamwongsatit, Kampon Kaeoket

**Affiliations:** 1 Department of Anatomy, Faculty of Medicine Siriraj Hospital, Mahidol University, Bangkok 10700, Thailand; 2 Semen Laboratory, Department of Clinical Sciences and Public Health, Faculty of Veterinary Science, Mahidol University, 999 Phuttamonthon 4 Rd., Salaya, Phuttamonthon, Nakhon Pathom 73170, Thailand; 3 Graduate Program in Biomedical Sciences, Faculty of Allied Health Sciences, Thammasat University, Rangsit Campus, Klong Luang, Pathumthani 12120, Thailand; 4 Department of Clinical Sciences and Public Health, Faculty of Veterinary Science, Mahidol University, 999 Phuttamonthon 4 Rd., Salaya, Phuttamonthon, Nakhon Pathom 73170, Thailand; 5 Laboratory of Bacteria, Veterinary Diagnostic Center, Faculty of Veterinary Science, Mahidol University, 999 Phuttamonthon 4 Rd., Salaya, Phuttamonthon, Nakhon Pathom 73170, Thailand

**Keywords:** artificial insemination, antimicrobial peptides, boar semen storage, *Escherichia coli*, gentamicin reduction, semen extenders, sperm quality, synthetic peptide PA-13

## Abstract

**Background and Aim:**

Bacterial contamination during liquid storage of boar semen negatively affects sperm quality and fertility outcomes, necessitating the routine use of antibiotics in semen extenders. However, increasing concerns regarding antimicrobial resistance have encouraged the development of alternative antimicrobial strategies. Synthetic antimicrobial peptides (AMPs) have demonstrated broad-spectrum antibacterial activity and may serve as potential substitutes or adjuncts to conventional antibiotics. This study aimed to evaluate the antimicrobial efficacy of the synthetic peptide PA-13 alone and in combination with reduced gentamicin concentrations against *Escherichia coli* isolated from boar semen, as well as its effects on semen quality during storage at 18°C.

**Materials and Methods:**

Two experiments were conducted. In Experiment I, fresh semen samples collected from seven healthy adult boars were diluted in Beltsville Thawing Solution supplemented with gentamicin (200 μg/mL, positive control), without antibiotics (negative control), or PA-13 at concentrations of 62.5, 31.25, and 15.625 μg/mL. Total bacterial counts were measured at 0, 24, 48, and 72 h of storage, while semen quality parameters, including sperm motility, viability, acrosomal integrity, and mitochondrial membrane potential, were evaluated on days 1, 3, and 5. In Experiment II, isolated *E. coli* was incubated with PA-13 alone or in combination with varying gentamicin concentrations, and bacterial growth was monitored over 24 h using optical density measurements.

**Results:**

PA-13 effectively inhibited bacterial proliferation in extended semen during the first 24 h of storage, with lower concentrations (15.625 and 31.25 μg/mL) showing greater antibacterial activity than the higher concentration (62.5 μg/mL). Semen quality parameters were comparable among groups on day 1; however, prolonged storage demonstrated that the highest PA-13 concentration negatively affected sperm motility and viability. Lower PA-13 concentrations preserved semen quality similar to that of the gentamicin-treated control. In Experiment II, combination treatments exhibited synergistic effects, with PA-13 at 3.906 μg/mL combined with gentamicin at 100 μg/mL inhibiting *E. coli* growth equivalently to gentamicin at 200 μg/mL alone.

**Conclusion:**

PA-13 effectively controls bacterial contamination in stored boar semen while maintaining semen quality at appropriate concentrations. Its combination with gentamicin enables a substantial reduction in antibiotic dosage without compromising antibacterial efficacy. These findings support the use of AMPs as alternative or complementary antimicrobial agents in semen extenders to reduce antibiotic use in the swine industry.

## INTRODUCTION

In response to the escalating global challenge of antimicrobial resistance (AMR), comprehensive “One Health” strategies have been proposed to reduce the overuse of conventional antibiotics [1]. Among these approaches, the use of antimicrobial peptides (AMPs) has emerged as a promising alternative for controlling bacterial contamination in swine production, with the potential to reduce or replace traditional antimicrobial agents in semen extenders and other veterinary applications, thereby limiting the transmission of antibiotic-resistant bacteria from animals to humans [1, 2]. Artificial insemination (AI) using liquid-stored boar semen is widely practiced in the swine industry due to its advantages in minimizing disease transmission, enhancing genetic improvement, and increasing both the quantity and quality of piglets produced [3, 4]. Bacterial contamination of boar semen adversely affects sperm quality and viability [2, 5, 6]. Moreover, insemination with contaminated semen may result in reproductive tract infections, including endometritis and abnormal vaginal discharge, as well as increased risks of embryonic or fetal loss in sows [5, 7].

Various Gram-positive and Gram-negative bacteria have been identified in fresh boar semen, including *Staphylococcus* spp., *Streptococcus* spp., *Escherichia coli*, *Klebsiella* spp., *Aeromonas* spp., *Pseudomonas* spp., *Proteus* spp., and *Providencia* spp. [8–11]. Antibiotics are routinely incorporated into boar semen extenders to inhibit bacterial contamination, prevent infection transmission to gilts and sows, and maintain spermatozoa function during storage [2, 6, 12].

AMPs have gained increasing attention as alternative antibacterial agents, particularly in response to the growing prevalence of antibiotic-resistant bacterial strains. They have been extensively studied as potential substitutes to reduce antibiotic usage, which has been reported to reach as high as 79.3 tons per year [2, 13]. The antibacterial mechanism of AMPs is primarily attributed to differences in electric charge between the peptides and bacterial membranes [2]. Several studies have examined the application of AMPs, including A-11, AP19, and BiF2_5K7K, as alternatives to antibiotics in boar semen extenders, demonstrating effective bacterial inhibition without compromising key semen quality parameters [14, 15].

PA-13, a synthetic AMP, has recently been identified as a promising alternative to conventional antimicrobial agents in boar semen extenders due to its ability to eliminate drug-resistant bacteria, particularly *E. coli* and *Pseudomonas aeruginosa* strains carrying resistance genes such as *int1* and *mcr-3*. This distinctive property of PA-13 has not been reported for A-11, AP19, or BiF2_5K7K [16, 17]. Currently, gentamicin is commonly added to boar semen extenders at concentrations ranging from 200 to 250 μg/mL to suppress bacterial contamination [2, 18, 19]. However, bacteria isolated from boar semen have shown increasing resistance to gentamicin and other antibiotics [11].

Although AMPs have attracted considerable attention as alternatives to conventional antibiotics in boar semen extenders, existing studies have mainly focused on their antibacterial activity alone or on short-term semen preservation outcomes. There remains limited evidence regarding the optimal concentrations of synthetic peptides that can effectively control bacterial contamination without compromising sperm quality during extended storage periods. In particular, information on the application of PA-13 within liquid-stored boar semen systems is scarce compared with previously investigated peptides such as A-11, AP19, and BiF2_5K7K. Furthermore, the potential synergistic interaction between AMPs and commonly used antibiotics, especially gentamicin, has not been sufficiently explored as a strategy to reduce antibiotic dosage while maintaining antimicrobial efficacy against prevalent semen contaminants such as *E. coli*. In addition, few studies have simultaneously evaluated microbial suppression alongside comprehensive semen quality parameters across multiple storage durations. These gaps limit the development of effective AMP-based approaches to address AMR while sustaining reproductive performance in AI programs.

Therefore, this study aimed to evaluate the antimicrobial effectiveness of PA-13 alone and in combination with reduced gentamicin concentrations against *E. coli* isolated from boar semen. Additionally, the study aimed to determine the effects of different PA-13 concentrations on key semen quality parameters during liquid storage at 18°C. By integrating bacterial inhibition outcomes with detailed semen quality assessments over time, this research sought to identify an optimized antimicrobial strategy that reduces antibiotic usage while maintaining semen functionality for sustainable swine reproductive management.

## MATERIALS AND METHODS

### Ethical approval

All procedures involving animals were conducted in accordance with the ARRIVE (Animal Research: Reporting of *In Vivo* Experiments) guidelines 2.0, the ethical principles outlined in the Animal for Scientific Purposes Act B. E. 2558 (Thailand), and the institutional policies of Mahidol University. The study protocol was reviewed and approved by the Institutional Animal Care and Use Committee of the Faculty of Veterinary Science, Mahidol University (FVS-MU-IACUC Protocol No. MUVS-2021-10-41). The corresponding animal use license No. U1-01281-2558 was obtained prior to commencement of the study.

Semen collection was performed using the well-established gloved-hand technique on healthy adult boars under routine farm management conditions. No sedation, restraint beyond standard handling, or any procedure likely to cause pain, suffering, or distress was required. The study involved only non-invasive sampling of ejaculates and did not involve any additional interventions, treatments, or euthanasia of animals.

### Study period and location

All boar semen samples were collected in May 2022, from three pig farms in Chainat, Chonburi, and Chachoengsao provinces.

### Peptide synthesis

As presented in Table 1, the peptide used in this study was previously synthesized and characterized for its physicochemical properties and antibacterial activity [16, 17]. Briefly, PA-13 was synthesized using solid-phase peptide synthesis with 9-fluorenylmethoxycarbonyl (Fmoc) chemistry and purified as trifluoroacetate salts by reversed-phase high-performance liquid chromatography (ChinaPeptides, Shanghai, China) [16, 17].

**Table 1:** Physicochemical properties of PA-13 [16, 17].

Peptide	Amino acid sequences	Number of amino acids	Molecular weight (g/mol)	Net charge	Percentage of hydrophobic residues	Purity
PA-13	KIAKRIWKILRRR	13	1736.25	+7	46%	95%

### Boar semen collection and preparation

Semen samples were collected from seven healthy adult Duroc, Landrace, and Large White boars aged between 1.5 and 3 years using the gloved-hand technique [20]. The sample size was determined using G-power software and approved by the Animal Care and Use Committee of the Faculty of Veterinary Science, Mahidol University. Only the sperm-rich fraction was retained after filtration through gauze. Ejaculates were used only when progressive motility was ≥70% and sperm concentration exceeded 100 × 10^6^ spermatozoa/mL [14, 15].

Fresh semen was diluted immediately at room temperature using Beltsville Thawing Solution (Minitube, Tiefenbach, Germany), either supplemented with gentamicin (200 μg/mL), without antibiotics, or with PA-13 at concentrations of 62.50, 31.25, and 15.625 μg/mL according to the minimum inhibitory concentration against *E. coli* [17]. Each group was adjusted to a final concentration of 3.0 × 10^9^ spermatozoa/100 mL. Diluted semen samples were stored at 18°C and assessed for total bacterial concentration at 0, 24, 48, and 72 h [14, 15]. Semen quality parameters were evaluated on days 1, 3, and 5 of storage.

### Total bacterial count

After incubation at 18°C, total bacterial concentration was determined using the spread plate technique. Samples were serially diluted tenfold in 0.85% normal saline solution (NaCl) [13, 14]. For each dilution, 100 μL was spread onto plate count agar (Difco, Nevada, USA) and incubated at 37°C for 48 h in duplicate. Visible colonies were counted and expressed as CFU/mL [14, 15].

### Semen quality parameters

Four semen quality parameters were analyzed after incubation at 18°C: sperm motility, sperm viability, sperm acrosomal integrity, and sperm with high mitochondrial membrane potential (MMP).

#### Sperm motility

Sperm motility was assessed using computer-assisted sperm analysis with AndroVision® (Minitube, Tiefenbach, Germany). Glass slides and stage temperature were maintained at 37°C. A 3 μL semen aliquot was placed in a counting chamber (Leja®, IMV Technologies, L’Aigle, Basse-Normandie, France). Five fields were examined per sample, with at least 600 sperm evaluated per analysis. Results were expressed as percentages of total and progressive motility [14, 15, 20].

#### Sperm viability

Sperm viability was evaluated using Ethidium homodimer-1 (EthD-1, E1169, Invitrogen, Waltham, MA, USA) and SYBR-14 (Live/Dead™ Sperm Viability Kit, Invitrogen). A mixture of 10 μL semen, 2.7 μL SYBR-14, and 10 μL EthD-1 was incubated at 37°C for 20 min. Subsequently, 5 μL of stained sample was placed on a glass slide, covered with a coverslip, and examined under a fluorescence microscope at 1000× magnification. A total of 200 sperm were classified as live or dead [14, 15, 20].

#### Sperm acrosomal integrity

Acrosomal integrity was assessed using fluorescein isothiocyanate-labeled peanut agglutinin (FITC-PNA). Semen samples (10 μL) were incubated with 10 μL EthD-1 at 37°C for 15 min. After fixation with 95% ethanol for 30 s, slides were stained with diluted FITC-PNA (1:10 in phosphate-buffered saline) and incubated at 4°C for 30 min in a moist chamber. After washing and air-drying, 200 sperm were examined under a fluorescence microscope and classified as having intact or damaged acrosomes [14, 15, 20–22].

#### Sperm with high MMP

MMP was analyzed using 5,5′,6,6′-tetrachloro-1,1′,3,3′-tetraethylbenzi-midazolyl-carbocyanine iodide (JC-1, T3168, Invitrogen). A mixture containing 2.4 mM PI solution, 3 μL JC-1 in dimethyl sulfoxide, and 50 μL semen was incubated at 37°C for 10 min. A total of 200 PI-negative sperm were evaluated at 400× magnification and classified as having low (green fluorescence) or high (yellow-orange fluorescence) membrane potential [14, 15, 20].

### Bacterial survival assay

An antibiotic-resistant *E. coli* strain was isolated from boar semen obtained from the Bacterial Laboratory, Veterinary Diagnostic Center, Faculty of Veterinary Science, Mahidol University, Thailand. The isolate was cultured in brain heart infusion broth and adjusted to the 0.5 McFarland standard (~10^8^ CFU/mL) using 0.85% NaCl.

For the assay, 48-well plates were prepared in triplicate, each containing 500 μL bacterial suspension diluted to 10^6^ CFU/mL in Mueller–Hinton broth (Difco, Reno, NV, USA). PA-13 and gentamicin solutions at different concentrations (Table 2) were added at 500 μL per well. Bacterial growth was monitored hourly for 24 h at 37°C by measuring OD_600_ using a microplate spectrophotometer (SPECTROstar Nano, BMG LABTECH, Ortenberg, Germany) after shaking for 10 s before each reading [14, 17].

**Table 2:** PA-13 and gentamicin concentrations used in the bacterial survival assay.

Group	PA-13 (μg/mL)	Gentamicin (μg/mL)
1	7.813	–
2	3.906	–
3	–	200
4	–	100
5	–	50
6	7.813	100
7	7.813	50
8	3.906	100
9	3.906	50
Growth control	–	–

### Statistical analysis

Statistical analyses were performed using PASW Statistics for Windows version 18.0 (SPSS Inc., Chicago, IL, USA). Normality was assessed using the Shapiro–Wilk test, and homogeneity of variance was evaluated using Levene’s test. Total bacterial concentration and semen quality data were log-transformed due to non-normal distribution. One-way analysis of variance was applied, followed by Duncan’s multiple range test for mean comparisons. Results are presented as means with 95% confidence intervals. A p-value < 0.05 was considered statistically significant. Descriptive statistics were applied for bacterial survival assay data.

## RESULTS

### Total bacterial count

Following sample collection, the total bacterial concentration of fresh boar semen was log 2.57 ± 0.41 CFU/mL. Bacterial concentrations in all experimental groups increased progressively with storage duration (Table 3). At 0 h, the negative control group exhibited significantly higher bacterial concentrations compared with both the PA-13-treated groups and the positive control group. At 24 h, bacterial levels in the negative control group remained significantly higher than those in the positive control group. Notably, all PA-13-treated groups demonstrated bacterial concentrations ranging from log 2.56 to log 3.02 CFU/mL, which were comparable to the positive control throughout the experimental period (Table 3). At identical incubation times, PA-13 at 62.5 μg/mL consistently exhibited higher bacterial concentrations than the lower PA-13 concentrations.

**Table 3:** Means ± SD of bacterial concentration (CFU/mL) (n = 7) at 0, 24, 48, and 72 h after incubation at 18°C.

Group	Peptide concentration (μg/mL)	0 h	24 h	48 h	72 h
BTS	–	1.63 ± 0.21^a^	3.04 ± 0.48^ b^	5.85 ± 1.27	8.67 ± 2.90
BTS + ABO	–	0.00 ± 0.00^ b^	0.00 ± 0.00^ a^	0.00 ± 0.00^ a^	1.17 ± 0.16^ a^
PA-13	62.5	0.82 ± 0.03^ b^	3.02 ± 1.56^ b^	8.54 ± 2.96	8.67 ± 2.96
PA-13	31.25	0.63 ± 0.18^b^	2.68 ± 0.25^ a, b^	8.55 ± 2.98	8.60 ± 2.96
PA-13	15.625	1.02 ± 0.07^ b^	2.56 ± 0.36^ a, b^	8.39 ± 2.81	8.42 ± 2.81

a,b Significant difference between PA-13 (62.5, 31.25, and 15.625 μg/mL) and negative and positive control groups at the same incubation time (*p* < 0.05). ABO: Antibiotic (gentamicin 200 μg/mL), BTS: Beltsville Thawing Solution, SD = Standard deviation.

### Semen quality parameters

**Figure 1:**
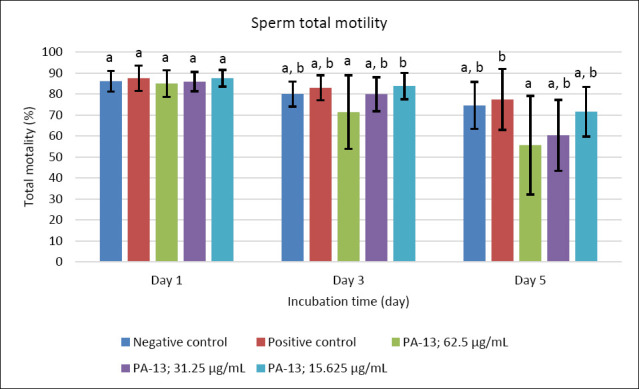
The effect of PA-13 on total motility on days 1, 3, and 5 after incubation at 18°C (Mean ± Standard error of the mean) (n = 7). a,b Significant difference between the PA-13 (62.5, 31.25, and 15.625 μg/mL) and negative and positive control groups at the same incubation time (p < 0.05).

**Figure 2:**
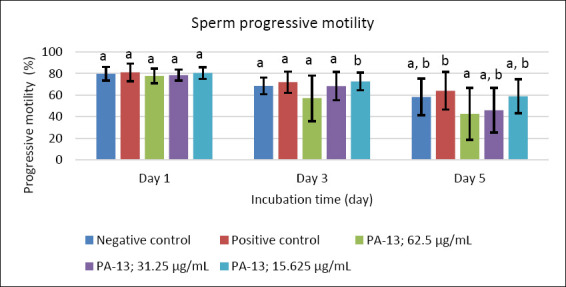
Effect of PA-13 on progressive motility on days 1, 3, and 5 after incubation at 18°C (Mean ± Standard error of the mean) (n = 7). a,b Significant difference between the PA-13 (62.5, 31.25, and 15.625 μg/mL) and negative and positive control groups at the same incubation time (p < 0.05).

**Figure 3:**
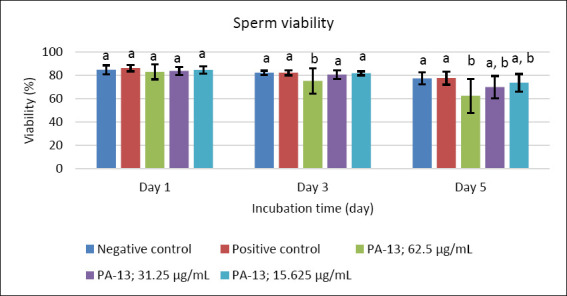
Effect of PA-13 on sperm viability on days 1, 3, and 5 after incubation at 18°C (Mean ± Standard error of the mean) (n = 7). a,b Significant difference between the PA-13 (62.5, 31.25, and 15.625 μg/mL) and negative and positive control groups at the same incubation time (p < 0.05).

**Figure 4:**
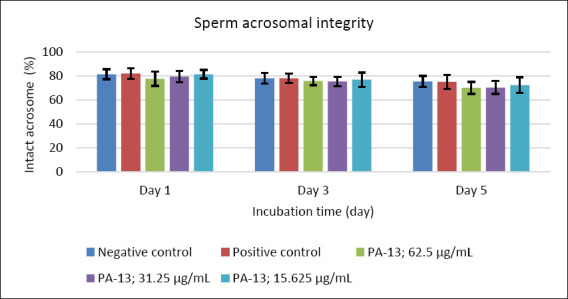
Effect of PA-13 on sperm acrosomal integrity on days 1, 3, and 5 after incubation at 18°C (Mean ± Standard error of the mean) (n = 7). This parameter was not statistically different between the PA-13 (62.5, 31.25, and 15.625 μg/mL) and the negative and positive control groups at the same incubation time.

**Figure 5:**
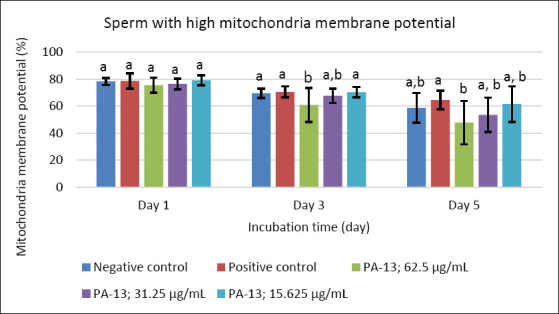
Effect of PA-13 on sperm with high mitochondrial membrane potential on days 1, 3, and 5 after incubation at 18°C (Mean ± Standard error of the mean) (n = 7). a,b Significant difference between the PA-13 (62.5, 31.25, and 15.625 μg/mL) and negative and positive control groups at the same incubation time (p < 0.05).

After supplementation with PA-13, the semen extender remained within standard specifications, with osmolality ranging from 305 to 316 mOsm/kg. Semen quality parameters declined with increasing storage time in all experimental groups, including the negative and positive controls (Figures 1–5). On day 1, no significant differences were observed among groups across all evaluated parameters (Figures 1–5).

On day 3, the greatest decline was observed in all parameters except sperm acrosomal integrity (Figure 4). Total and progressive motility demonstrated similar trends, with the highest PA-13 concentration (62.5 μg/mL) causing the most pronounced reduction in semen quality compared with lower concentrations, particularly 15.625 μg/mL and the positive control (Figures 1 and 2). Sperm viability and sperm with high MMP showed no significant differences among groups, except in the PA-13 62.5 μg/mL group, which exhibited a significantly lower sperm viability rate (Figures 3 and 5).

By day 5, all semen quality parameters, except sperm acrosomal integrity, were more adversely affected in PA-13-treated groups than in the negative and positive controls (Figures 1, 2, 3, and 5), while acrosomal integrity remained unaffected (Figure 4). The highest PA-13 concentration consistently resulted in the greatest deterioration in semen quality compared with lower concentrations (Figures 1–5).

### Bacterial survival assay

**Figure 6:**
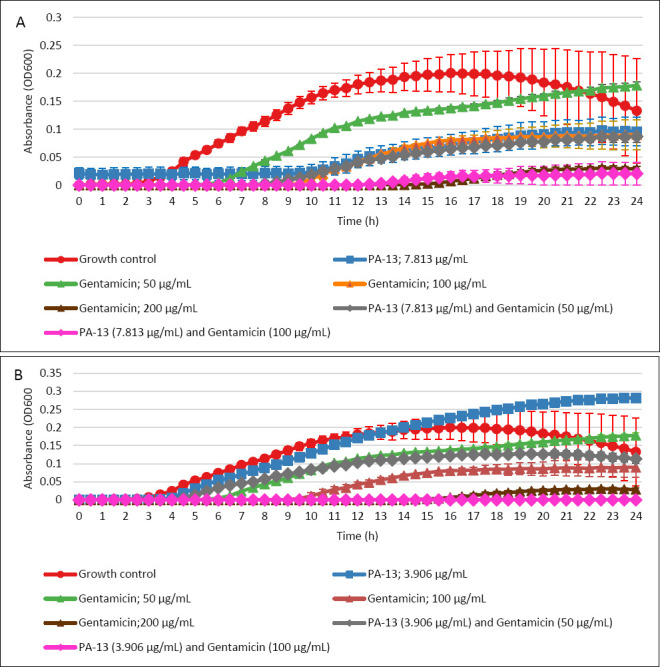
Growth curves of *Escherichia coli* over 24 h (mean ± Standard error of the mean), incubated with PA-13 at (A) 7.813 μg/mL and (B) 3.906 μg/mL in combination with different gentamicin concentrations. Gentamicin at 100 μg/mL (A: pink line, B: pink line) and PA-13 at (A) 7.813 and (B) 3.906 μg/mL inhibited *E. coli* growth in an effective manner equivalent to that of gentamicin at 200 μg/mL (A: brown line, B: brown line) alone.

Figure 6 illustrates the growth curves of *E. coli* over 24 h. The combination of gentamicin at 100 μg/mL with PA-13 at 7.813 μg/mL (Figure 6A) and 3.906 μg/mL (Figure 6B) effectively inhibited bacterial growth, demonstrating efficacy comparable to gentamicin at 200 μg/mL alone. Additionally, the combination of PA-13 at 7.813 μg/mL with gentamicin at 50 μg/mL suppressed bacterial growth to a similar extent as PA-13 at 7.813 μg/mL alone or gentamicin at 100 μg/mL alone (Figure 6A).

## DISCUSSION

### Antibacterial effectiveness of PA-13 in stored boar semen

The present findings demonstrated that replacing gentamicin with PA-13 in boar semen extenders during the first 24 h of storage effectively suppressed bacterial growth in semen doses intended for AI. PA-13 at concentrations of 31.25 and 15.625 μg/mL reduced bacterial levels to ranges considered acceptable for maintaining farm production efficiency within the initial 24 h [21]. Boar semen contaminated with *E. coli* at concentrations exceeding log 3.54 CFU/mL has been reported to negatively impact reproductive performance, resulting in reduced litter size [23]. Additionally, contamination with *P. aeruginosa* at levels ranging from log 4 to log 6 CFU/mL has been associated with decreased fertilizing capacity [24]. In the present study, PA-13 at 31.25 and 15.625 μg/mL effectively controlled bacterial growth after 24 h of storage at 18°C at levels below those known to impair sperm quality. Since total bacterial concentration was assessed rather than species-specific thresholds, direct comparisons with fertility impairment limits for individual bacterial species could not be performed.

The short-term semen extender used in this study is capable of preserving semen quality for up to 3 days following dilution, although storage may be extended to 5 days [22]. Accordingly, semen quality was evaluated on days 0, 1, 3, and 5 to confirm that semen parameters were maintained within acceptable ranges in accordance with manufacturer recommendations [15].

### Concentration-dependent antibacterial activity of PA-13

Lower concentrations of PA-13 exhibited greater antibacterial efficacy in boar semen compared with higher concentrations. This observation aligns with previous leakage assay findings, which indicated that higher PA-13 concentrations caused less disruption to *E. coli* cell membranes than lower concentrations [17]. Greater leakage of bacterial genetic material was observed at lower PA-13 concentrations, suggesting enhanced membrane permeabilization and antibacterial activity [17]. Similarly, in the present study, the highest PA-13 concentration did not produce the strongest antibacterial effect within the semen extender environment.

This phenomenon may be attributed to electrostatic repulsion that occurs at higher peptide concentrations, which can reduce the binding affinity of AMPs to bacterial membranes, thereby decreasing membrane disruption and bacterial cell lysis [25]. As previously reported, accumulation of positively charged peptides with a net charge of +7 may diminish bactericidal efficacy at elevated concentrations compared with lower doses [25].

### Mechanisms of AMP interaction with bacterial and animal cell membranes

AMPs exert antibacterial activity primarily through electrostatic interactions with bacterial cell membranes. While animal cell membranes, including boar spermatozoa, possess a negatively charged inner membrane surface that interacts weakly with AMPs, bacterial membranes exhibit strong negative charges on their outer surfaces, facilitating AMP binding and subsequent membrane disruption [26, 27]. This interaction ultimately leads to bacterial cell death [26, 28, 29]. Structural damage to bacterial membranes following AMP exposure has been confirmed through transmission electron microscopy and scanning electron microscopy analyses [17].

### Influence of hydrophobicity on AMP toxicity

The cytotoxic effects of AMPs on animal cells are closely related to their hydrophobicity levels. Peptides with higher hydrophobicity have been shown to induce greater damage to animal cells compared with those exhibiting lower hydrophobicity [27, 30]. Comparative studies of A11 and AP-19, which differ in hydrophobicity, demonstrated that AP-19 caused greater sperm toxicity due to its higher hydrophobic properties [14]. Hydrophobicity plays a crucial role in the selectivity of AMPs between bacterial and animal cells, as peptides with higher hydrophobic content more readily penetrate animal cell membranes, resulting in cellular damage [14]. Furthermore, hydrophobicity is positively correlated with peptide charge, with highly charged AMPs often exhibiting increased antimicrobial activity alongside elevated cytotoxicity [31]. Modifications in AMP structure or amino acid sequence have been suggested as strategies to reduce toxicity toward animal cells while preserving antimicrobial effectiveness [31, 32].

### Synergistic effects of PA-13 and gentamicin

Combining PA-13 with gentamicin significantly reduced the effective antibiotic concentration to 100 μg/mL while maintaining antibacterial activity against *E. coli* comparable to that achieved with 200 μg/mL gentamicin alone. Similar synergistic effects have been reported for *P. aeruginosa* when treated with SAAP-18 in combination with tetracycline [33]. Such combinations may reduce the toxicity associated with high concentrations of both AMPs and antibiotics, although further studies are required to evaluate their long-term effects on semen quality.

These findings support the concept that AMPs can be effectively combined with conventional antibiotics to overcome individual limitations and enhance antimicrobial efficacy [33, 34]. Consistent with previous research, the present study reinforces the potential of AMPs as viable alternatives or adjuncts to antibiotics in boar semen extenders, enabling reduced antibiotic usage [12, 15].

From a practical perspective, the application of AMPs offers significant benefits for reducing antibiotic use within the swine industry, particularly in AI units. In line with the “One Health” framework, AMP-based strategies may contribute to limiting the spread of antibiotic-resistant bacteria while promoting sustainable livestock production [1].

Although PA-13 demonstrated effective bacterial inhibition and maintained semen quality in vitro, its impact on sow reproductive performance following AI has not yet been evaluated. Therefore, further in vivo studies are necessary to assess fertility outcomes and validate the practical application of PA-13 in commercial swine production systems.

## CONCLUSION

This study demonstrated that the synthetic AMP PA-13 effectively suppressed bacterial growth in stored boar semen during the initial 24 h of preservation, with lower concentrations (15.625 and 31.25 μg/mL) providing superior antibacterial efficacy compared with higher concentrations. These concentrations successfully maintained bacterial levels below thresholds associated with impaired reproductive performance, while preserving key semen quality parameters throughout short-term storage. In addition, the combination of PA-13 with gentamicin exhibited a synergistic effect, enabling a 50% reduction in antibiotic concentration while achieving antibacterial activity comparable to higher gentamicin doses against *E. coli*.

The application of PA-13 as an alternative or adjunct antimicrobial agent in boar semen extenders offers a practical strategy for reducing antibiotic usage in AI programs. By maintaining microbial control while preserving semen quality, PA-13 may contribute to improved reproductive efficiency and support antimicrobial stewardship efforts within the swine industry. The synergistic combination approach further enhances feasibility by lowering reliance on conventional antibiotics without compromising antibacterial performance.

A major strength of this study lies in its integrated evaluation of both antimicrobial efficacy and compre-hensive semen quality parameters over multiple storage durations. The use of antibiotic-resistant *E. coli* provides clinically relevant insights into real-world contamination scenarios. Additionally, the investigation of combination therapy with gentamicin highlights a novel approach for minimizing antibiotic concentrations.

Despite promising in vitro outcomes, this study assessed total bacterial load rather than species-specific fertility thresholds within semen doses. Furthermore, reproductive performance following AI using PA-13-treated semen was not evaluated, limiting direct translation of laboratory findings to field fertility outcomes.

Future studies should focus on in vivo fertility trials to assess sow reproductive performance following AI with PA-13-supplemented semen. Long-term storage effects, potential impacts on offspring health, and broader antimicrobial activity against diverse semen-associated bacteria, including *P. aeruginosa*, should also be explored. Optimization of AMP formulations to minimize cytotoxicity while maximizing antimicrobial activity represents an additional area of interest.

Overall, PA-13 represents a promising AMP-based strategy for controlling bacterial contamination in boar semen extenders while reducing dependence on conventional antibiotics. Through its effective antimicrobial action, compatibility with semen quality, and synergistic interaction with gentamicin, PA-13 offers a sustainable alternative aligned with “One Health” objectives to mitigate AMR in swine reproductive management.

## DATA AVAILABILITY

The supplementary data can be made available from the corresponding author upon request.

## AUTHORS’ CONTRIBUTIONS

Kr. K. performed the experiments, collected and analyzed the data, and wrote the first draft of the manuscript. R. A. synthesized the antimicrobial peptide, designed and supervised the study, and edited the manuscript. P. C. designed and supervised the study, performed semen quality analysis, and collected the data. N. N. designed and supervised the study, conceived the idea, investigated the study, analyzed the data, and edited the manuscript. K. K. conceptualized and supervised the study and clinical trial, edited the manuscript, secured funding, and administered the project. All authors have read and approved the final version of the manuscript.
